# Indirect Inlay Method

**Published:** 1915-06

**Authors:** Henry W. Gillett


### INDIRECT INLAY METHOD.

BY HENRY W. GILLETT, D.M.D.

It is not only my habit to use the indirect system in the production of nearly all the inlays which go through my hands, but it is my belief that in this way I do my patients greater service, and do it more easily for myself than would be the case if I were to use the direct system.

The chief reason for my choice has to do with the amalgam die, and the fact that such die, or duplicate, of the cavity is readily obtainable at the second transfer from the cavity, and that this die serves as a substitute for the cavity in all further work, enabling the operator to correct such deficiencies and errors in the subsequent steps as it is important to have corrected.

I hold that the statement of the advocates of the direct process that the indirect introduces more chance of error, is based upon mistaken premises. I believe that the process of taking an impression of the cavity in a suitable modeling compound, is as little subject to error as is the step of forming the wax core in the cavity, and that the making of the amalgam die is *less* subject to error, than almost any other step in either inlay process.

If these claims be well founded, then we have, in case of accurate work, a die which is very close indeed to being a true replica of the cavity. When we have such a replica but two steps from the cavity on which the procedures needed for correcting subsequent errors may be carried out, it seems to me self-evident that the operator is in a more advantageous position for carrying them out than he is when they must be done on the tooth itself. Especially do I feel this to be the case when, as is my own custom, the operator desires to use a gold that is harder than 24-K gold, and which is, therefore, more difficult to manipulate in making the required corrections. I believe that all castings, regardless of the process used, or the skill of the operator, require some of these corrections, and that the man who talks about reproducing *absolutely* his wax core, or about getting an *absolute* fit of the cavity, is talking a manifest absurdity.

It is claimed by some advocates of the direct system, that the indirect system always results in a product smaller than the cavity. This I can neither affirm nor deny. I can only express the hope that it is a correct statement because I believe it to be a distinct advantage. My reason is, that this fact, if it be a fact, interferes in no way with the effective anchoring of the inlay. Assuming correct cavity formation, this slight reduction in size will not unfavorably affect the anchorage of the inlay in such a manner as to make possible its dislodgement by stress in any other direction than the one opposite to that from which it was inserted, nor can it, with an accurate amalgam replica of the cavity at hand, introduce any real difficulty in the way of establishing a close fit of the margin.

These two essential points having been provided for, it would then be my choice to have a liberal layer of cement between the gold and the cavity wall. I prefer to regard my gold inlays as tight stoppers covering cement fillings.

I feel that it is the aim of the present-day practitioner to do something more than preserve the tooth from decay, or even to restore its proximal contour. It is necessary, in order to live up to the standards of today, that wherever possible the efficiency of a broken-down molar or bicuspid, as a masticator, shall be restored. I feel that but few operators will dispute the dictum that the two materials, which we use as fillings, with which this end can best and most readily be accomplished, are the gold inlay and amalgam. A few may hold that porcelain should be mentioned and the gold crown is not to be forgotten. And when contemplating restoring the efficiency of a broken-down molar I of course include the reproduction of its occlusal surface normal to the mouth in which it stands.—Extracted from article in *Journal of Allied Sciences* for March.

